# GSDME deficiency leads to the aggravation of UVB-induced skin inflammation through enhancing recruitment and activation of neutrophils

**DOI:** 10.1038/s41419-022-05276-9

**Published:** 2022-10-01

**Authors:** Yujie Chen, Ni Lian, Sihan Chen, Ta Xiao, Yangying Ke, Yiqun Zhang, Changjun Song, Yong Yang, Song Xu, Heng Gu, Xu Chen

**Affiliations:** 1 Jiangsu Key Laboratory of Molecular Biology for Skin Diseases and STIs, Institute of Dermatology, Chinese Academy of Medical Sciences & Peking Union Medical College, 210042 Nanjing, China; 2 Key Laboratory of Basic and Translational Research on Immune-Mediated Skin Diseases, Chinese Academy of Medical Sciences, Nanjing, China

**Keywords:** Cell death, Cell death and immune response, Inflammation

## Abstract

Gasdermin E (GSDME)-mediated pyroptosis is induced in keratinocytes of UVB-challenged skin. The role of GSDME in UVB-caused skin damage remains unknown. To explore the role of GSDME in UVB-induced skin inflammation. We compared differences in skin appearance, histological features, keratinocyte death modalities, infiltration of immune cells, and levels of some inflammatory cytokines between *Gsdme*^−/−^ mice and wild type (WT) mice after UVB exposure. We explored whether keratinocytes contribute to GSDME deficiency-caused aggravation of UVB-induced skin inflammation in GSDME knockdown keratinocyte cultured in vitro and keratinocyte-specific *Gsdme* conditional knockout mice. We used anti-Ly6G antibody to deplete neutrophils and explore their role in UVB-caused skin damage. Skin damage and neutrophils infiltration were aggravated in UVB-challenged *Gsdme*^−/−^ mice, compared with UVB-challenged WT mice. Apoptosis and necroptosis, which were initiated together with GSDME-mediated pyroptosis in UVB-challenged WT mice, were not enhanced in UVB-challenged *Gsdme*^−/−^ mice. Neutrophils activation indicators and its recruiting cytokines were increased in skin tissue of UVB-challenged *Gsdme*^−/−^ mice. However, GSDME knockdown did not lead to the further increase of mRNA and secretion of TNF-α and IL-6 in UVB-challenged keratinocytes. Skin damage was not aggravated in UVB-challenged *Gsdme* cKO mice. Neutrophils depletion alleviated UVB-caused skin damage in WT mice and *Gsdme*^−/−^ mice, and eliminated its aggravation in *Gsdme*^−/−^ mice. This study demonstrates that GSDME plays a restrictive role in UVB-induced skin damage through inhibiting excessive recruitment and activation of neutrophils in the immune microenvironment in UVB-caused skin inflammation. However, keratinocytes might not contribute to this restrictive function.

## Introduction

Exposure to ultraviolet (UV) radiation leads to extensive influences on biology and pathology of the human being. Because of the higher irradiated intensity and lower penetrated ability in different UV wavelengths, UVB could cause more significant disturbances on skin homeostasis, especially the epidermis. Exposed to different dose and cumulative time of UV radiation, human skin could appear sunburn, skin inflammation, photoaging, photocarcinogenesis and other disorders [1]. Cellular responses of keratinocytes in the epidermis in response to UV-caused damage plays a crucial role in the development of these diseases. Among the UV-related cellular mechanisms, UV-induced keratinocyte death is one of core events contributing skin damage caused by UV radiation.

In contrast to biologically uncontrolled accidental cell death (ACD), regulated cell death (RCD) is controlled under structured signaling cascades and defined molecular mechanisms [2]. Recently, RCDs were divided into different molecularly definitions of terms, for example, intrinsic apoptosis, extrinsic apoptosis, pyroptosis, necroptosis, and other modalities [3]. For a long time, apoptosis was identified to be the type of keratinocyte death in UV-challenged skin [4]. Recently, we reported that UVB radiation induces typical pyroptosis morphological change and activation of pyroptosis executor gasdermin E (GSDME) in human keratinocytes [5]. Vats et al found that ferroptosis is triggered in keratinocytes of UVB-challenged skin [6]. Therefore, it can be speculated that UV-caused keratinocyte death is non-single RCD modality.

Epidermis is the major target tissue of UVB radiation due to limited penetration of UVB rays. As inflammatory response is a crucial inherently protective mechanism, skin inflammation is promptly triggered after epidermal damage inflicted by UVB irradiation. It was found that the auto-amplification loop exists between RCD and inflammation. For instance, interplay between RCD and inflammation initiates an auto-amplification loop in the hypoxic conditions, for instance, hypoperfused cells in organs [7]. During RCD process, damage-associated molecular patterns (DAMPs) and proinflammatory cytokines could be released from necroptotic cells to activate the immune cells. Furthermore, immune cells infiltrating in damaged tissue trigger RCD through various pathways such as IFN-γ or TNF signaling [7]. However, it remains unclear how keratinocytes participate in regulation of skin inflammation through executing RCDs in UV-challenged skin.

Although the ultimate goal of different types of RCD is self-destruction, distinct nature of cell necroptotic process leads to different regulatory effects on inflammatory responses (non-inflammatory or proinflammatory), and dictates physiological and pathological outcomes in turn. Pyroptosis is gasdermin protein-mediated RCD with membrane pore formation. Accompanied with formation of gasdermin-mediated membrane pore, proinflammatory cytokines (IL-1β and IL-18 in gasdermin D (GSDMD)-mediated pyroptosis) and other intracellular proteins are released to participate in the inflammation process [8]. Recently, Xia et al. reported that *Gsdme* deficiency mice exhibit a low level of acute kidney injury and inflammatory responses [9]. Therefore, we speculate that GSDME-mediated pyroptosis might be involved in the regulation of skin inflammatory response after UVB exposure. Here, we explored the role of GSDME in skin inflammation of UVB-challenged mice.

## Materials and methods

### Cell culture

Human keratinocyte cell line HaCaT cells (China Center for Type Culture Collection) were cultured in Dulbecco’s modified Eagle’s medium containing 10% fetal bovine serum (Gibco, Invitrogen Corp., Carlsbad, CA, USA). HaCaT cells have been authenticated using STR profiling.

### Reagents and antibodies

Primary antibodies against DFNA5/GSDME N-terminal (#ab215191), phospho-MLKL (Ser345 and Ser358, #ab196436 and #ab187091), CD4 (#ab183685), CD8 (#ab217344), MPO (#ab208670), Histone H3 (citrulline R2 + R8 + R17, #ab5103), matrix metalloproteinase 9 (MMP-9, #ab283575), neutrophil elastase (NE, #ab68672), wide spectrum Cytokeratin (#ab9377), Ly6G (#ab238132) were purchased from Abcam (Cambridge, MA, USA). Primary antibodies against PARP (#9532), MLKL (#37705, #14993), Caspase 3 (#9662), F4/80 (#70076), Perforin (#44865), GAPDH (#5174) and anti-rabbit IgG HRP-linked secondary antibodies (#7074) were purchased from Cell Signaling Technology (Danvers, MA, USA). The primary antibody against C5b-9 (#bs-2673R) was purchased from Bioss Inc (Beijing, China).

### Animal study

C57BL/6JGpt mice (WT), *Gsdme*^−/−^ (*Gsdme* KO) mice (B6/JGpt-*Gsdme*^em8Cd566^/Gpt), B6/JGpt-*Gsdme*^*em1Cflox*^/Gpt and B6/JGpt-H11^em1Cin(hKRT14-iCre)^/Gpt mice were purchased from GemPharmatech (Nanjing, Jiangsu, China). Keratinocyte-specific *Gsdme* cKO mice (Krt14^Cre/+^-*Gsdme*^flox/flox^, GemPharmatech) and control mice (Krt14^+/+^-*Gsdme*^flox/flox^, GemPharmatech) were offspring mice through intercrossing from B6/JGpt-*Gsdme*^em1Cflox^/Gpt and B6/JGpt-H11^em1Cin(hKRT14-iCre)^/Gpt mice. All mice used were 5–8 weeks of age and of 15–25 g. Mice were randomly allocated to experimental groups. At 24 h before UVB exposure, we shaved the dorsal skin of all mice. For inducing acute skin inflammation, mice were exposed to 430 mJ/cm^2^ UVB, as described previously [10]. All mice were sacrificed at indicated times after UVB exposure, and dorsal skin samples were collected. Skin samples were immersed into 2 mg/ml dispase II (Sigma-Aldrich, St. Louis, MO, USA) to separate the epidermis. We obtained approval of animal studies from Medical Ethics Committee in the Institute of Dermatology, Chinese Academy of Medical Sciences (2019-DW-002), and the Institutional Animal Care and Use Committee at Nanjing Medical University (IACUC-2101043).

### Scores of UVB-induced skin damage

Skin manifestations of erythema, edema and skin destruction were respectively scored as 0 (None), 1 (Mild), 2 (Moderate), and 3 (Severe) to evaluate damage level of UVB-challenged skin. Details are shown in Table 1. One investigator who was blind to the specific group evaluated skin damage and recorded the scores.

**Table 1:** Evaluation of UVB-induced skin damage.

Score	Erythema	Edema	Skin destruction
0 (None)	Similar to the unexposed skin		
1 (Mild)	Pink	Mild swelling	Erosion
2 (Moderate)	Red	Slightly harden	Erosion and exudation
3 (Severe)	Dark red	Harden	Ulcer

### Propidium iodide (PI) staining

PI staining assay was performed as described previously [10] in the frozen skin tissue section.

### Immunofluorescence assay

Preparation of skin sample was performed as previous description [10]. Tissues were subsequently incubated in primary antibodies at 4 °C overnight. Next, secondary antibody goat anti-rabbit IgG (Alexa FluorTM 488, A-11008; Alexa FluorTM 647, A-21244, Thermo Fisher, Waltham, MA, USA) was used.

Tissue was incubated in Hoechst 33342 (Cell Signaling Technology) for visualizing the nucleus. PI/RNase staining solution (Cell Signaling Technology) was used to visualize dead cells if needed.

Positive staining cells were counted in 3 different views in the same magnification from sections of each sample. The means of each sample were used for statistical analysis.

### Western blotting assay

Preparation of protein samples and western blotting assay were performed as previous description [10]. We used the ImageJ software to quantify the density of interested protein bands.

### Immunohistochemistry study

Preparation of skin tissue samples and immunohistochemistry assay were performed as previously described [10].

We randomly took 6 different views from tissue sections. By ImageJ software, positive staining cells were counted, and the dimension of each view was measured. Positive staining cells per square millimeter represented infiltrating levels of immune cells in skin tissue.

### ELISA

Secretion of CXCL-1, LCN2, TNF-α, IL-1β, and IL-6 was determined using ELISA kits. ELISA kits included CXCL-1, LCN2, IL-1β (above from Beijing 4 A Biotech, Beijing, China), TNF-α (Absin Bioscience, Shanghai, China), and IL-6 (Thermo Fisher).

### RNA extraction and quantitative real-time reverse transcription PCR

Total RNA of tissue or cells was extracted by RNAex Pro Reagent (Accurate Biology, Hunan, China). RNA was reversely transcribed to cDNA using Evo M-MLVRT kit (Accurate Biology). Table 2 lists primer sequences. Quantitative PCR was performed using SYBR Green Premix Pro Taq HS qPCR Kit (Accurate Biology). Relative levels of interesting mRNA were calculated by 2(-ΔΔ C(T)) method for statistical analysis.

**Table 2:** Primer sequences in this study.

Primer (mice)	Sequences (5’→3’)
Cxcl1-m-F	TGGCTGGGATTCACCTCAA
Cccl1-m-R	TGGCTATGACTTCGGTTTGG
Il1b-m-F	TTCCTGAACTCAACTGTGAAATGC
Il1b-m-R	TGTTGATGTGCTGCTGCGAG
Lcn2-m-F	TCGCTACTGGATCAGAACATTTG
Lcn2-m-R	GAACTGGTTGTAGTCCGTGGTG
Tnfa-m-F	ATGAGCACAGAAAGCATGATCC
Tnfa-m-R	AGGCTGAGACATAGGCACCG
Il6-m-F	GACTTCCATCCAGTTGCCTT
Il6-m-R	ATGTGTAATTAAGCCTCCGACT
Ifng-m-F	TCAACAACCCACAGGTCCAG
Ifng-m-R	CAGCAGCGACTCCTTTTCC
Gapdh-F	TGTGTCCGTCGTGGATCTGA
Gapdh-R	TTGCTGTTGAAGTCGCAGGAG
**Primer (human)**	**Sequences (5’→3’)**
CXCL-1-h-F	CACCCCAAGAACATCCAAAGT
CXCL-1-h-R	TGTCACTGTTCAGCATCTTTTCG
IL-1β-h-F	TGATGGCTTATTACAGTGGCAA
IL-1β-h-R	TAGTGGTGGTCGGAGATTCG
LCN2-h-F	AGACAAAGACCCGCAAAAGAT
LCN2-h-R	AAACAGGACGGAGGTGACATT
TNFα-h-F	CTTCCAGCTGGAGAAGGGTG
TNFα-h-R	CCCAAAGTAGACCTGCCCAG
IL-6-h-F	AGTGAGGAACAAGCCAGAGC
IL-6-h-R	GGTCAGGGGTGGTTATTGCA
IFNγ-h-F	TGGAGACCATCAAGGAAGACAT
IFNγ-h-R	GCGACAGTTCAGCCATCACT
GAPDH-F	GCACCGTCAAGGCTGAGAAC
GAPDH-R	TGGTGAAGACGCCAGTGGA

### GSDME gene knockdown

We transfected pGLVH1/GFP + Puro plasmids (C06003) containing GSDME short hairpin RNA (shRNA) or nonsense control (NC) shRNA (GenePharma, Suzhou, Jiangsu, China) into HaCaT cells. NC shRNA sequence: 5’-TTCTCCGAACGTGTCACGT-3’; GSDME shRNA sequence: 5’-GCAGAAGTGTGTGATCTCTGA-3’. Efficiently transfected HaCaT cells were selected by puromycin incubation.

### Neutrophil depletion

To deplete neutrophils, mice were injected intraperitoneally with an initial 400 μg and followed by 100 μg every 2 days (for a total of five times) for anti-Ly6G monoclonal antibody (BP0075-1, BioXCell, Lebanon, NH, USA). Control mice were injected with equal amounts of isotype control antibodies. On 1 day after the 4th injection, all mice were exposed to UVB radiation.

### Single-cell suspensions preparation

The skin tissue of mice was dissected and washed by cold PBS. Tissue fragments was incubated in lysis buffer in 37 °C for 1 h. Lysis buffer contained 3 ml DMEM, 10 U/ml Dnase I (Absin), 0.2% Collagenase I (17100017, Thermo Fisher), and 5 mM CaCl_2_. Then, the supernatant was collected and neutralized with PBS. The residual tissue fragments were re-incubated in lysis buffer in the same condition. Finally, the supernatant collected from two replications was filtered together to be subjected to flow cytometry analysis.

### Flow cytometry

7-Amino-Actinomycin D (7-AAD) (#559925, BD, Franklin Lake, NJ, USA) was used to stain dead cells. FITC Rat Anti-Mouse CD45 (#553080, BD), PE-Cy™7 Rat Anti-CD11b antibody (#552850, BD), APC Rat Anti-Mouse Ly6G (#560599, BD), and Ly6G/Ly-6C (Gr-1) monoclonal antibody, APC (#RB6-8C5, eBioscience, Waltham, MA, USA) were used to incubate cells.

For the analysis of neutrophils in skin tissue, single-cell samples and peripheral blood cells were incubated with 7-AAD, CD45, CD11b, and Ly6G. FSC-H/FSC-A was used to identify single cells. 7-AAD was used to identify live cells. And the percentage of neutrophils (CD45^+^CD11b^+^Ly6G^+^) was calculated from CD45^+^ cells.

To analyze the efficiency of neutrophil depletion, peripheral blood of mice was collected and incubated with CD11b and Gr-1. Flow cytometry was performed using BD FACSerse^TM^ (BD). FlowJo software was used to analyze the data.

### Statistical analysis

When variances are equal between groups, Student’s *t* test (for two groups) and one-way ANOVA (the differences between more than two groups were compared by Bonferroni correction) were used for statistical analysis. When variances are unequal between groups, Kruskal–Wallis test followed by Dunn’s post hoc test was used. We obtained data for statistical analysis from at least three independent experiments. The variance was similar between the groups that were being statistically compared. We did not use any statistical method to predetermine the sample size. “*n* = ” represents how many biological replicates we used in our experiments, as shown in the figures and figure legends. All data in this study were presented as mean ± SD. *P* < 0.05 was taken as evidence of statistical significance.

## Results

### *Gsdme*^−/−^ mice presented aggravated skin inflammation after UVB exposure

**Fig. 1:**
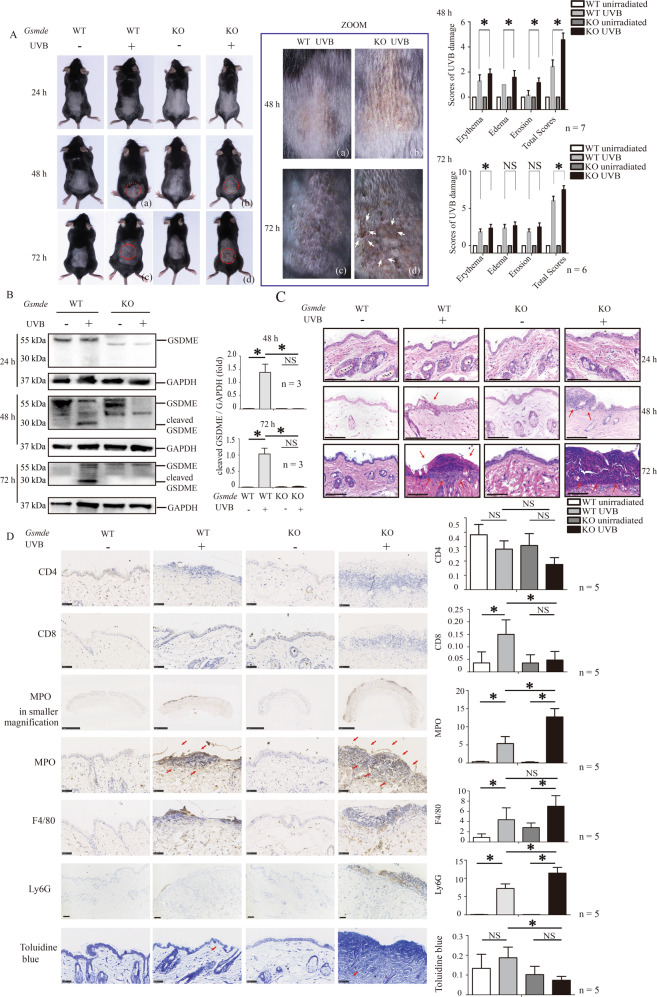
*Gsdme*^−/−^ mice present aggravated skin inflammation after UVB exposure. **A** WT mice (C57BL/6JGpt) and *Gsdme*^-/-^ (*Gsdme* KO) mice were exposed or unexposed to UVB radiation (430 mJ/cm^2^) on dorsal skin. Photos showed skin appearances of mice at 24, 48, or 72 h after UVB exposure. White arrows indicated skin destruction. Skin damage caused UVB radiation was evaluated by scores. The details of scores were described in “Materials and methods”. **B** GSDME cleavage was detected in epidermis lysate of WT or *Gsdme*^−/−^ mice through western blotting assay at 24, 48, and 72 h after irradiation. Quantification of the interested protein bands was shown (*n* = 3). **C** Representative images of H&E staining present the histological features. Scale bar presents 100 μm. The red arrow indicates epidermal necrosis. **D** Images of immunohistochemistry study showed the infiltrating levels of CD4^+^ T cells, CD8^+^ T cells, MPO^+^ cells, Ly6G^+^ cells, F4/80^+^ macrophages, and toluidine blue positive mast cells in skin tissue of WT or *Gsdme*^−/−^ mice at 48 h after UVB exposure. Positive cell counts per square millimeter were used to perform quantitative analysis (*n* = 5). The scale bar presents 50μm, except for the scale bar (1 mm) in the images of MPO in smaller magnification. The red arrow indicates positive cells. *P < 0.05. NS nonsense.

In our previous study [5], we found that UVB radiation induces GSDME cleavage in cultured keratinocytes. Therefore, we detected whether GSDME is cleaved in the keratinocytes of UVB-challenged mice (Fig. 1A, B). We compared the difference of skin damage between *Gsdme*^−/−^ and WT mice after UVB exposure (Fig. 1A). We found that GSDME cleavage occurred in epidermis lysates of WT mice at 48 and 72 h after exposure (Fig.1B. Meanwhile, we had not observed GSDME cleavage in UVB-challenged *Gsdme*^−/−^ mice (Fig. 1B). UVB-challenged WT mice manifested acute skin inflammation features including erythema, edema and erosion (Fig. 1A). Interestingly, *Gsdme*^−/−^ mice presented more severe UVB-caused skin manifestations than WT mice at 48 and 72 h after exposure (Fig. 1A). UVB-challenged *Gsdme*^−/−^ mice presented more severe histological features including epidermis thickens, intraepidermal edema, and epidermis necrosis than that of WT mice (Fig. 1C). We compared the difference of immune cells’ infiltration such as CD4^+^ T cells, CD8^+^ T cells, F4/80^+^ macrophages, MPO^+^ cells and Ly6G^+^ cells between *Gsdme*^−/−^ mice and WT mice (Fig. 1D).

### UVB radiation simultaneously induces apoptosis, pyroptosis, and necroptosis

**Fig. 2:**
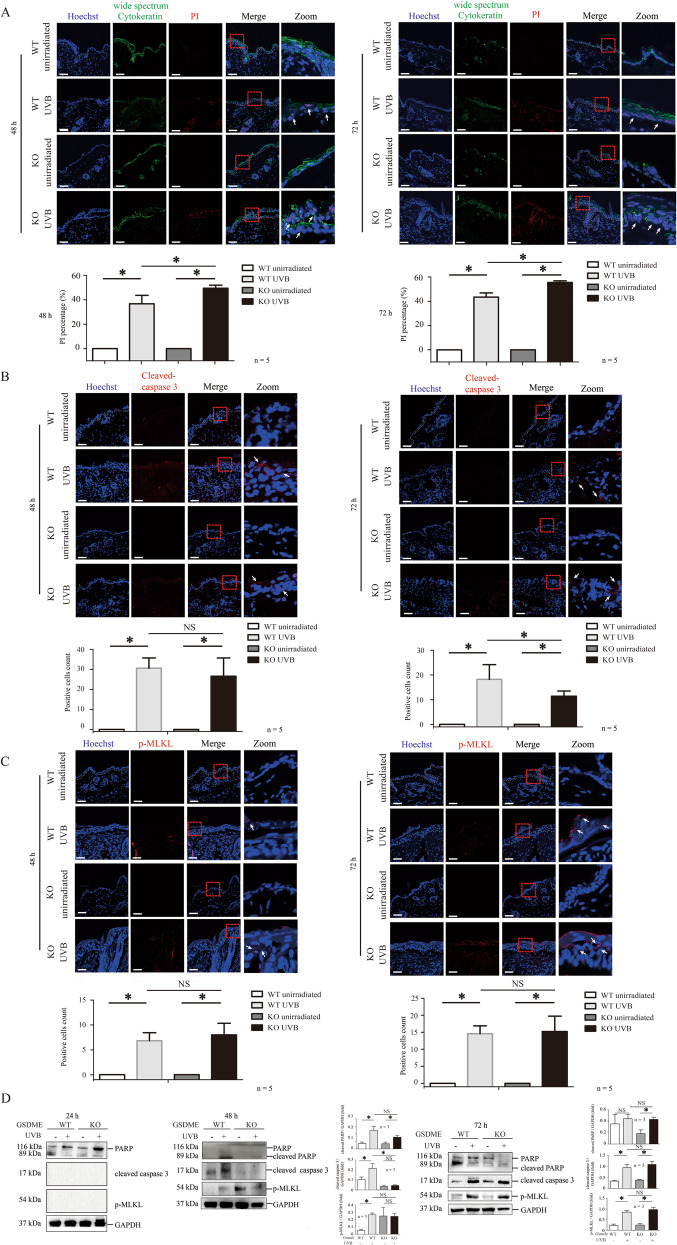
Pyroptosis, apoptosis, and necroptosis were triggered simultaneously in UVB-challenged mice. **A** The level of keratinocyte death was determined by PI staining. Wide spectrum Cytokeratin was used to indicate keratinocytes. Scale bar represents 100 μm, *n* = 5. **B**, **C** Caspase 3 (**B**), and phosphorylation at Ser345 site of MLKL (**C**) were detected by immunofluorescence assay. The nucleus was stained by Hoechst (*n* = 5). Scale bar represents 100 μm. **D** PARP, caspase 3, and phosphorylation at Ser345 site of MLKL were detected by western blotting assay in WT or *Gsdme*^−/−^ mice at 24, 48, and 72 h after UVB exposure. Quantification of the interested protein bands was shown (*n* = 3). *P < 0.05. NS nonsense.

Considering that UVB-challenged *Gsdme*^−/−^ mice presented more severe epidermal necrosis than UVB-challenged WT mice, we compared keratinocyte death level through PI staining assay. We found that keratinocyte death was increased in UVB-challenged *Gsdme*^−/−^ mice (Fig. 2A). Considering that epidermis necrosis was more intense in UVB-challenged *Gsdme*^−/−^ mice, we speculated that other RCDs might be induced alternatively when the pyroptosis cannot be engaged. Therefore, we detected molecular indicators of apoptosis and necroptosis. GSDME protein level was increased in the epidermis of UVB-challenged WT mice, compared with unchallenged WT mice. The GSDME increase induced by UVB cannot be observed in *Gsdme*^−/−^ mice, indicating GSDME deficiency (Supplementary Fig. S1). We observed that cleaved-caspase 3 (apoptosis indicator) and MLKL phosphorylation (necroptosis indicator) were increased in the epidermis of both WT mice and *Gsdme*^−/−^ mice after exposure (Fig. 2B, C). Moreover, cleaved-PARP (apoptosis indicator) and MLKL phosphorylation were increased in the epidermal lysate of UVB-challenged both WT mice and *Gsdme*^−/−^ mice (Fig. 2D). These findings demonstrate that apoptosis, pyroptosis and necroptosis are engaged simultaneously in UVB-challenged mice. Importantly, cleavage of PARP and caspase 3 and MLKL phosphorylation were not increased in UVB-challenged *Gsdme*^−/−^ mice, compared with UVB-challenged WT mice (Fig. 2B–D). Therefore, these findings indicate that epidermis necrosis aggravation in UVB-challenged *Gsdme*^−/−^ mice was not caused by the alternate increase of apoptosis and necroptosis.

### Neutrophils activation is enhanced in UVB-challenged *Gsdme*^−/−^ mice

To explore why keratinocyte death was increased in UVB-challenged *Gsdme*^−/−^ mice, we detected some indicators of immune cells associated cell death.

**Fig. 3:**
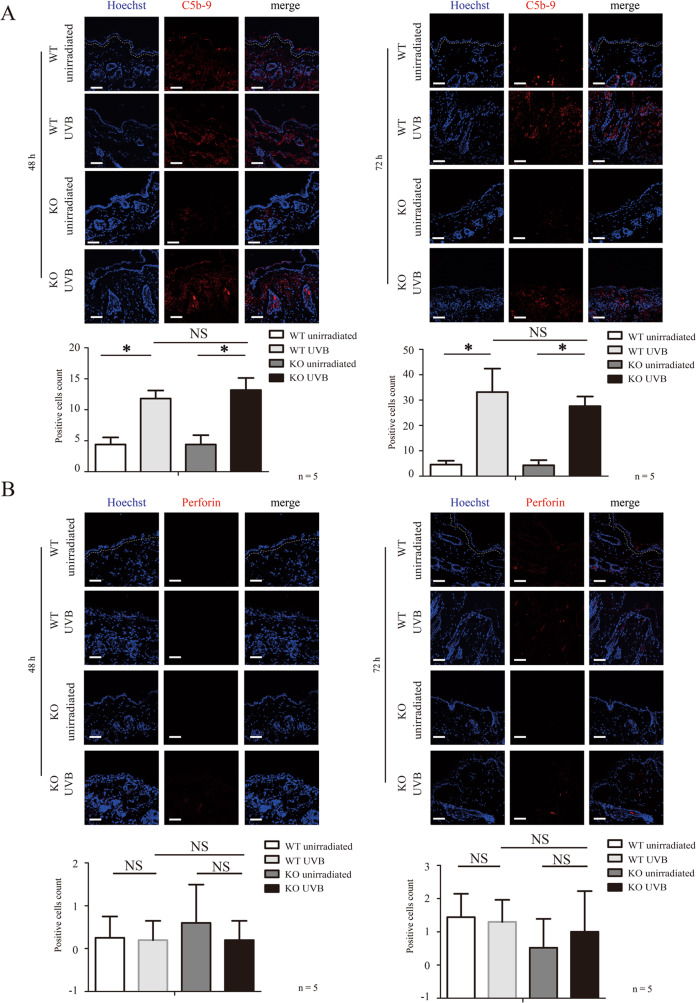

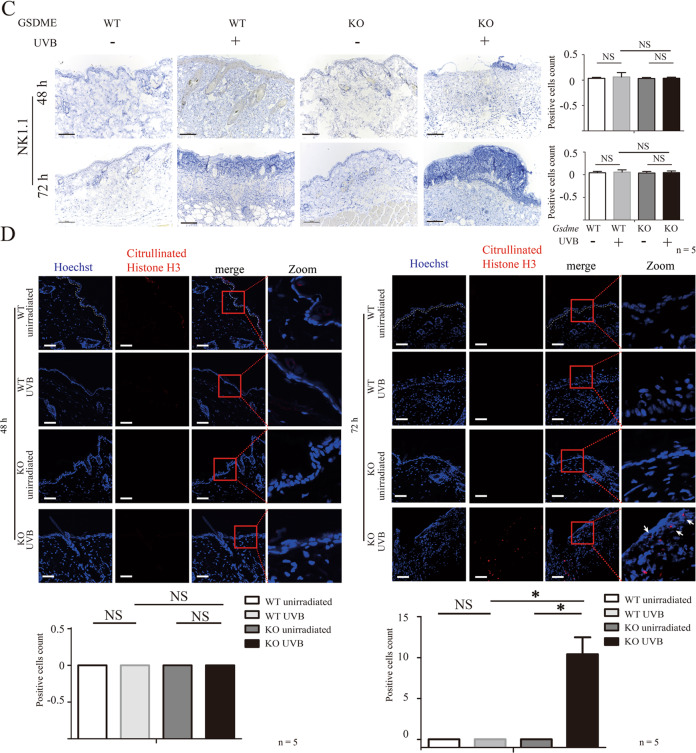
Membrane attack complex C5b-9 and perforin do not contribute to the increase of keratinocyte death of UVB-challenged *Gsdme*^−/−^ mice. **A**, **B** Membrane attack complex C5b-9 (**A**) and perforin (**B**) were detected in skin tissue at 48 and 72 h after UVB exposure by immunofluorescence assay (*n* = 5). The nucleus was stained by Hoechst. Scale bar represents 100 μm. **C** NK1.1^+^ NK cells were detected in skin tissue at 48 and 72 h after UVB exposure by immunohistochemistry assay (*n* = 5). The scale bar represents 100 μm. **D** The neutrophil extracellular traps indicator, Citrullinated Histone H3 was detected in skin tissue at 48 and 72 h after UVB exposure by immunofluorescence assay (*n* = 5). The nucleus was stained by Hoechst. The scale bar represents 100 μm. NS nonsense.

Membrane attack complex (MAC) is formed by series of complement components, including C5b, C6, C7, C8, and C9. MAC-mediated cell lysis is one consequences of complement system activation [11]. We observed an increase of C5b-9 deposition in skin tissue of WT mice and *Gsdme*^−/−^ mice after exposure (Fig. 3A), indicating complement system activation. We did not observe the difference in C5b-9 deposition between two groups (Fig. 3A). Perforin is one pore-forming protein, and it is mainly generated and released by natural killer cells and cytotoxic T lymphocytes [12]. When immunological reaction is activated, perforin is released and mediates target cell death through the formation of a transmembrane pore [12]. We had not observed an increase in perforin expression in UVB-challenged *Gsdme*^−/−^ and WT mice (Fig. 3B), suggesting that perforin might not contribute to UVB-induced keratinocyte death. We had not observed a difference of natural killer (NK) cells’ indicator NK1.1 between UVB-challenged and unchallenged WT mice (Fig. 3C). NK.1.1^+^ NK cells were not increased in UVB-challenged *Gsdme*^−/^− mice, compared with UVB-challenged WT mice (Fig. 3C).

Activated neutrophils release decondensed chromatin fibers to form neutrophil extracellular traps (NETs) [13]. Citrullinated histone H3 is a biomarker to detect NETs [13, 14]. In Fig. 1D, we showed that neutrophils infiltration was increased in UVB-challenged *Gsdme*^−/−^ mice, compared with UVB-challenged WT mice. Citrullinated histone H3 level was increased in UVB-challenged *Gsdme*^−/−^ mice but not in UVB-challenged WT mice (Fig. 3D), although the latter also presented neutrophils infiltration (Fig. 1D). These findings indicate that neutrophils might be over-activated in the skin inflammatory microenvironment of UVB-challenged *Gsdme*^−/−^ mice.

### Enhanced immune microenvironment promotes neutrophils activation in UVB-challenged *Gsdme*^−/−^ mice

**Fig. 4:**
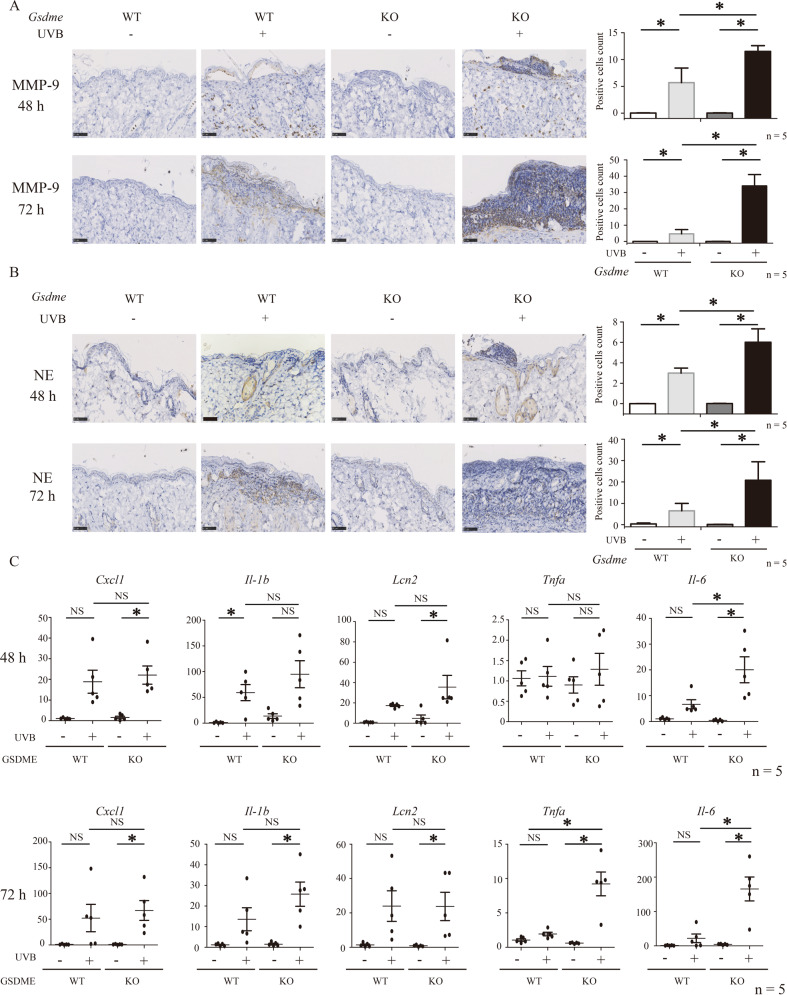
Neutrophils activation indicators MMP-9 and neutrophil elastase are increased in UVB-challenged *Gsdme*^−/−^ mice. **A**, **B** The protein levels of MMP-9 (**A**) and neutrophil elastase (NE) (**B**) were detected in skin tissue at 48 and 72 h after UVB exposure by immunohistochemistry assay (*n* = 5). Scale bar represents 50 μm. **C** The mRNA levels of Cxcl1, Il1b, Lcn2, Tnfα, and Il6 in whole skin lysate at 48 and 72 h after UVB exposure. GAPDH served as the reference gene. *n* = 5. *P < 0.05. NS nonsense.

To verify the enlarged activation of neutrophils, we detected neutrophils activation indicators, MMP-9 and NE as previous study [15]. Protein levels of MMP-9 and NE were increased in UVB-challenged both WT and *Gsdme*^−/−^ mice (Fig. 4A, B). In accordance to the findings of MPO^+^ cells count, Ly6G^+^ cells count and citrullinated histone H3 detection, UVB-challenged *Gsdme*^−/−^ mice presented increases of MMP-9 and NE, compared with UVB-challenged WT mice (Fig. 4A, B). These findings demonstrate that GSDME gene deficiency leads to an excessive increase of neutrophils’ infiltration and activation in UVB-challenged skin.

We determined mRNA levels of CXCL-1 [16], IL-1β [17], LCN2 [18], TNF-α [19], and IL-6 [20], which all are related to neutrophils recruitment, in skin tissue. The mRNA levels of TNF-α (at 72 h after exposure) and IL-6 (at 48 and 72 h after exposure) were elevated in UVB-challenged *Gsdme*^−/−^ mice, compared with UVB-challenged WT mice (Fig. 4C). These findings indicate that UVB-challenged *Gsdme*^−/−^ mice exhibited a stronger immune microenvironment for recruiting neutrophils than UVB-challenged WT mice.

### GSDME of keratinocyte might not contribute to the enhancement of neutrophils’ recruitment and activation in UVB-challenged *Gsdme*^−/−^ mice

**Fig. 5:**
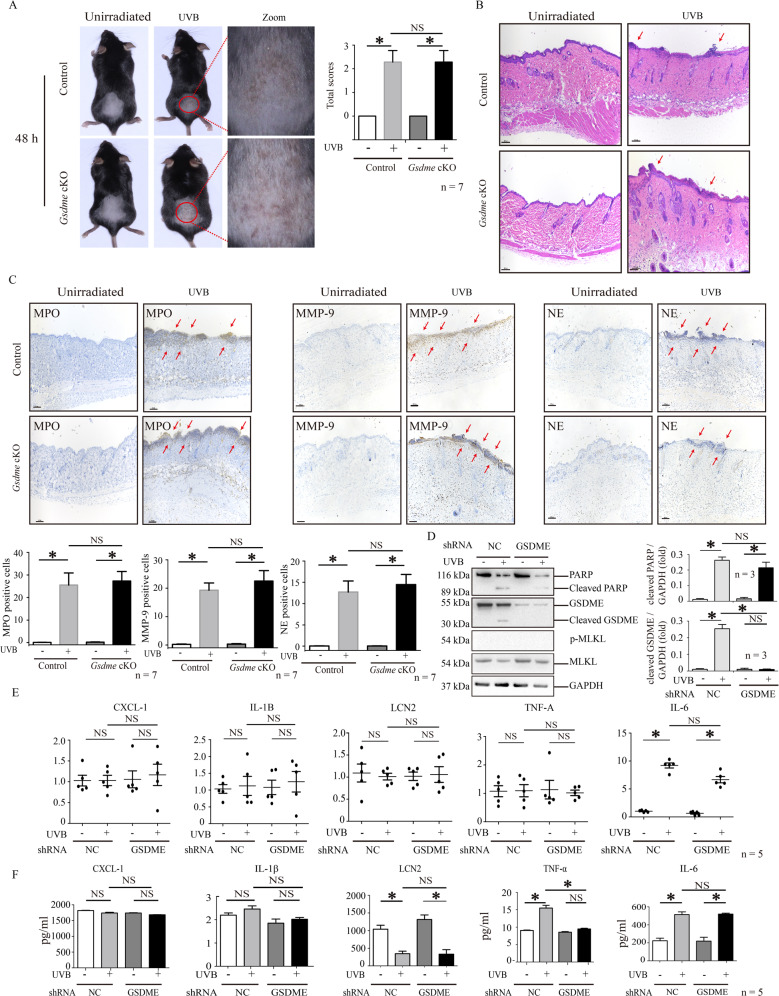
Keratinocyte might not contribute to the enhancement of neutrophils’ recruitment and activation in UVB-challenged *Gsdme*^−/−^ mice. **A** Keratinocyte-specific *Gsdme* cKO mice and control mice were exposed or unexposed to 430 mJ/cm^2^ UVB radiation on dorsal skin. Images showed the skin appearance at 48 h after exposure. Skin damage was evaluated by scores. The details of scores were described in “Materials and methods”. **B** Representative images of H&E staining present the histological features. Red arrow indicates epidermal necrosis. Scale bar represents 100 μm. **C** MPO, MMP-9, and NE were detected in skin tissue *Gsdme* cKO mice and control mice at 48 h after UVB exposure. Positive cell counts per square millimeter were used to perform quantitative analysis. The red arrow indicates positively marked neutrophils. The scale bar represents 100 μm. *P < 0.05. NS nonsense. **D** GSDME cleavage, PARP cleavage, and phosphorylation at Ser 358 site of MLKL were detected by western blotting assay in HaCaT with or without GSDME knockdown at 12 h after 50 mJ/cm^2^ UVB exposure. Quantification of the interested protein bands was shown (*n* = 3). **E** The mRNA levels of CXCL-1, IL-1β, LCN2, TNF-α, and IL-6 were determined by real-time quantitative reverse transcription PCR in HaCaT cells with or without GSDME knockdown at 12 h after 50 mJ/cm^2^ UVB exposure (*n* = 5). GAPDH served as the reference gene. **F** The secretion in cell culture supernatants of CXCL-1, IL-1β, LCN2, TNF-α, and IL-6 were determined by ELISA at 12 h after 50 mJ/cm^2^ UVB exposure (*n* = 5).

Considering that UVB radiation cannot induce GSDME-mediated pyroptosis in keratinocytes of *Gsdme*^−/−^ mice, we speculate that keratinocytes might contribute to enhancing recruitment and activation of neutrophils in UVB-challenged *Gsdme*^−/−^ mice. We compared the difference in skin appearance between UVB-challenged keratinocyte-specific *Gsdme* cKO mice (Krt14^Cre/+^-*Gsdme*^flox/flox^) and UVB-challenged control (Krt14^+/+^-*Gsdme*^flox/flox^) mice. GSDME deficiency in keratinocytes was confirmed (Supplementary Fig. S2a). We found that cKO mice exhibited similar skin damage appearance (Fig. 5A), inflammatory histological features (Fig. 5B), and levels of MPO, MMP-9, and NE as control mice after exposure (Fig. 5C). These findings validate that GSDME deficiency in keratinocytes do not alternately enhance skin damage and neutrophil activation after UVB exposure. To verify this speculation, we determined cytokines and mediators secreting from keratinocytes after UVB exposure, and explored the difference between GSDME knockdown and non-knockdown keratinocytes. We verified that GSDME protein was significantly reduced in knockdown HaCaT cells (Supplementary Fig. S2b). We confirmed that UVB cannot induce GSDME cleavage in knockdown HaCaT cells (Fig. 5D). We found that PARP cleavage was not increased in knockdown HaCaT cells after exposure (Fig. 5D), indicating that apoptosis is not enhanced when pyroptosis cannot be operated. However, we had not observed MLKL phosphorylation increase in knockdown and non-knockdown HaCaT cells after exposure (Fig. 5D), suggesting that UVB cannot initiate necroptosis in HaCaT cells cultured in vitro. In accordance with the results of study in vivo (Fig. 2), these findings validate that keratinocytes do not alternately enhance apoptosis and necroptosis after exposure when pyroptosis cannot be operated.

We found that both mRNA and secretion of CXCL-1, IL-1β, LCN2, TNF-α, and IL-6 were not increased in GSDME knockdown HaCaT cells than non-knockdown after exposure (Fig. 5E, F). These data indicate that GSDME deficiency in keratinocytes might not contribute to the increase of cytokines and mediator production for recruiting more neutrophils into UVB-caused skin inflammatory microenvironment.

### Treatment of monoclonal antibody against Ly6G inhibits the aggravation of UVB-caused skin damage in *Gsdme*^−/−^ mice

**Fig. 6:**
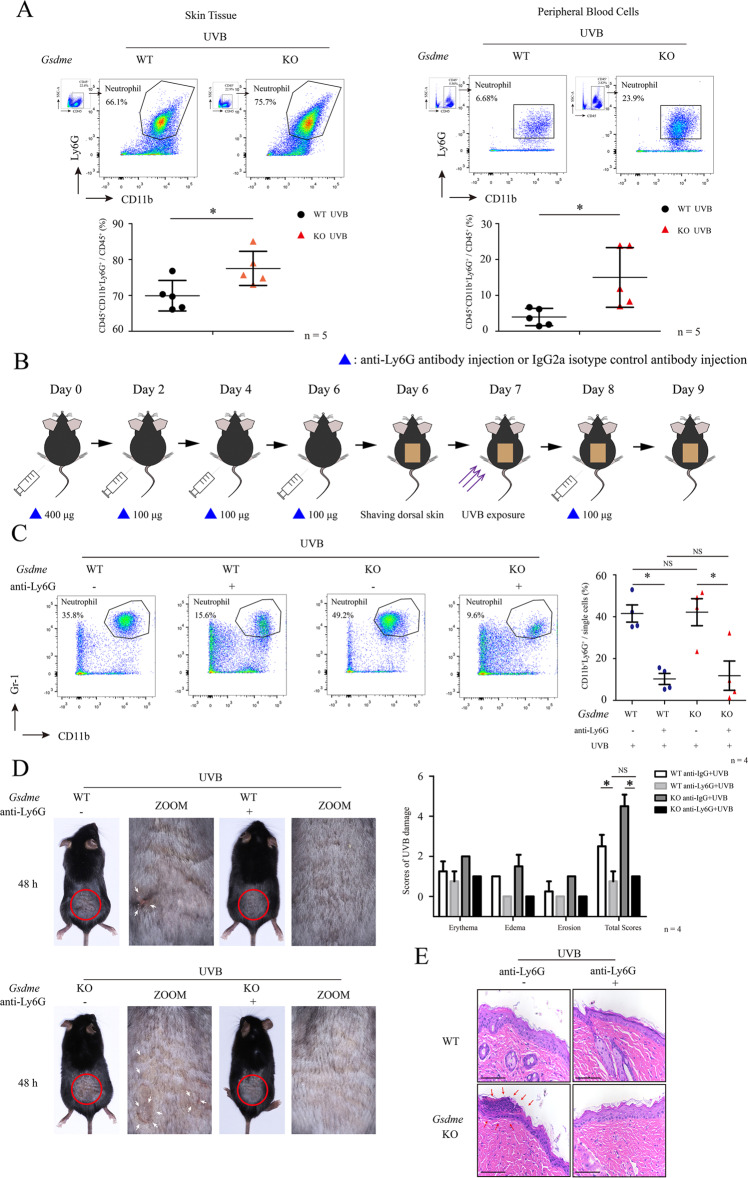

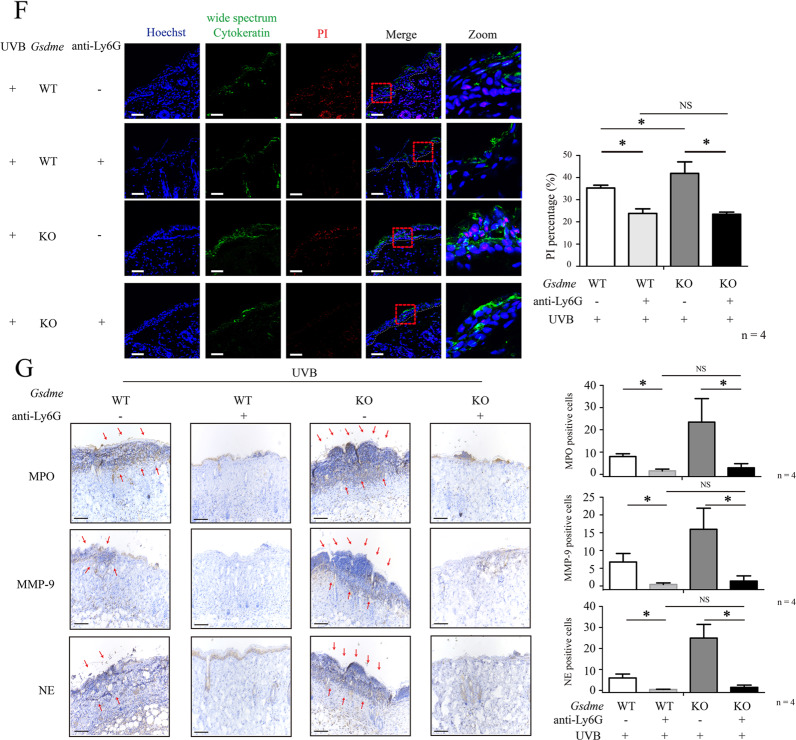
Depleting neutrophils eliminates the aggravation of UVB-caused skin damage in *Gsdme*^−/−^ mice. **A** Recruitment of neutrophils in the skin or peripheral blood at 72 h after UVB exposure were analyzed by flow cytometry. The frequency of CD11b^+^Ly6G^+^ cells in CD45^+^ gated live cells were used for quantitative analysis (*n* = 5). Detailed gating strategy was shown in Supplementary Fig. S5. **B** The flowchart shows the process in the experiment of UVB-challenged WT mice and *Gsdme*^−/−^ mice with anti-Ly6G injection. **C** Representative dot plots of CD11b^+^Gr-1^+^ neutrophils in peripheral blood of different groups of mice. Quantification analysis was performed by FlowJo software. **D** Images showed the skin appearance at 48 h after UVB exposure. Skin damage was evaluated by scores. The details of scores were described in “Materials and methods”. The white arrow indicates epidermal destruction. **E** Representative images of H&E staining present the histological features. Red arrow indicates epidermal necrosis. Scale bar presents 100 μm. **F** Level of keratinocyte death was determined by PI staining. Wide spectrum Cytokeratin was used to indicate keratinocytes. The scale bar represents 100 μm, *n* = 4. **G** MPO, MMP-9, and NE were detected in skin tissue through immunohistochemistry assay. Scale bar represents 100 μm. *P < 0.05. NS nonsense.

To further confirm whether GSDME deficiency leads to the over-recruitment of neutrophils, we measured the ratios of neutrophils (CD45^+^CD11b^+^Ly6G^+^ cells) versus CD45^+^ cells in skin tissue by flow cytometry in WT mice and *Gsdme*^−/−^ mice at 72 h after UVB exposure. We found that the ratios of UVB-challenged *Gsdme*^−/−^ mice were increased, validating the over-recruitment of neutrophils (Fig. 6A). To explore whether GSDME deficiency leads to the increased production of neutrophils in UVB-challenged *Gsdme*^−/−^ mice, we measured the ratios of neutrophils versus CD45^+^ cells in peripheral blood samples. We found that the ratios were increased in peripheral blood of UVB-challenged *Gsdme*^−/−^ mice, compared with UVB-challenged WT mice (Fig. 6A). These findings indicate that GSDME deficiency might lead to an increase of production of neutrophils after UVB exposure.

As a previous study [21], we used anti-Ly6G antibody to deplete Ly6G^+^ neutrophils and explore their role in the aggravation of UVB-caused skin damage in *Gsdme*^−/−^ mice. In UVB-challenged *Gsdme*^−/−^ and WT mice, we determined levels of CD11b^+^Gr-1^+^ (Gr-1 here was used to avoid false negative signals) cells in peripheral blood after anti-Ly6G antibody injection. We validated that CD11b^+^Gr-1^+^ cells were reduced in UVB-challenged *Gsdme*^−/−^ and WT mice after anti-Ly6G injection, compared with those injected by lgG2a isotype control (Fig. 6B). There was no difference of neutrophils between UVB-challenged *Gsdme*^−/−^ and WT mice with anti-Ly6G injection, as well as between two group mice with control injection (Fig. 6C). Depleting neutrophils decreased scores of UVB-caused skin damage in either *Gsdme*^−/−^ mice or WT mice, compared with those with control injection (Fig. 6D). There was no difference of UVB-caused skin damage scores between *Gsdme*^−/−^ mice and WT mice (Fig. 6D), suggesting that depleting neutrophils eliminated skin damage aggravation in UVB-challenged *Gsdme*^−/−^ mice. Histologically, epidermal necrosis was alleviated in UVB-challenged *Gsdme*^−/−^ mice and UVB-challenged WT mice after anti-Ly6G injection (Fig. 6E). Depleting neutrophils decreased the percentage of PI-positive keratinocytes in the epidermis in *Gsdme*^−/−^ mice and WT mice, compared with those with control injection (Fig. 6F), There was no difference of PI-positive keratinocytes between UVB-challenged *Gsdme*^−/−^ and WT mice with anti-Ly6G injection, indicating that neutrophils depletion eliminates the aggravation of epidermis necrosis in *Gsdme*^−/−^ mice (Fig. 6F). Counts of MPO^+^, MMP-9^+^, and NE^+^ cells were reduced in UVB-challenged *Gsdme*^−/−^ and WT mice after anti-Ly6G injection (Fig. 6G). These findings demonstrate that neutrophils contribute to the aggravation of UVB-caused skin damage in *Gsdme*^−/−^ mice.

## Discussion

Our study demonstrated that GSDME plays a restrictive role on inhibiting excessive inflammatory reaction through restraining infiltration and activation of neutrophils in UVB-challenged skin.

We found that UVB triggers simultaneously apoptosis, GSDME-mediated pyroptosis and necroptosis in keratinocytes. Vats et al. reported that ferroptosis, apoptosis and pyroptosis can be detected in UVB-challenged keratinocyte [6]. Our study also indicated that UVB-caused keratinocyte death is not a simple process of one certain RCD, but is operated in multiple RCD modalities. Vats et al. had not observed the necroptosis initiation indicator, phosphorylation of MLKL and RIPK3 in UVB-challenged keratinocyte [6]. We also cannot observe MLKL phosphorylation in UVB-challenged keratinocytes in vitro. However, we found that MLKL phosphorylation was increased in keratinocytes in UVB-challenged mice (Fig. 2). We speculate that UVB-induced infiltration of immune cells, which are major source to secret proinflammatory mediators and cytokines, might induce keratinocyte necroptosis. Thus, without promotion of UVB-activated immune cells, necroptosis cannot be initiated in cultured keratinocyte cultured in vitro after UVB exposure.

RCD pathway which is contemporaneously engaged with apoptosis, pyroptosis, and necroptosis was recently identified to be PANoptosis [22–24]. GSDMD-mediated and GSDME-mediated pyroptosis were observed to be triggered simultaneously in Karki et al.’s study [23]. However, GSDME-mediated pyroptosis was not reported to be initiated in PANoptosis without GSDMD activation. Karki et al. reported that co-treatment of TNF-α and IFN-γ initiates PANoptosis and leads to lethal cytokine storm in mice [23]. In our observation, although apoptosis, pyroptosis (GSDME-mediated) and necroptosis were simultaneously triggered in keratinocytes of UVB-challenged mice, inflammation was localized in skin tissue and IFN-γ was not elevated (Figs. 1 and 4C and Supplementary Fig. S3). Combined with the findings that UVB cannot simultaneously trigger these three RCD in vitro, thus, we speculate that mechanism of apoptosis, pyroptosis (GSDME-mediated) and necroptosis in keratinocytes of UVB-challenged mice are different from PANoptosis triggered by TNF-α and IFN-γ.

Recent studies indicated that PANoptosis is activated through the formation of PANoptosome [22, 25–27]. PANoptosome was currently identified to be consist of RIPK1, PYCARD, NLRP3, and caspase 8 [27]. RIPK3, caspase 6, ZBP1, and caspase 1 were also involved in initiation of PANoptosis [28]. Wang et al. reported that GSDME-mediated pyroptosis and apoptosis shared upstream regulator, caspase 3 [29]. We observed that the cleavage of GSDME is accompanied by the cleavage of caspase 3 in UVB-challenged keratinocytes [5]. However, there is currently no evidence that caspase 3 is involved in PANoptosis process. Therefore, we speculated that the three RCD in UVB-challenged keratinocytes might be mediated by a pathway different from PANoptosis.

Caspase 1-dependent pyroptosis induced by various pathological challenging is acknowledged as inflammatory cell death [30, 31]. Inflammasome-mediated caspase 1 activation leads to GSDMD N-terminal production through GSDMD cleavage. GSDMD N-terminal results in pore formation of the cell membrane and intensive release of proinflammatory cytokines such as IL-1β and IL-18 [31]. Therefore, we once speculated that UVB-triggered keratinocyte pyroptosis plays a proinflammatory role in UVB-induced inflammation. However, skin inflammation was not relieved in UVB-challenged keratinocyte-specific *Gsdme* cKO mice, and even worsened in *Gsdme*^−/−^ mice. These findings suggest that GSDME-mediated keratinocyte pyroptosis might not have a proinflammatory effect similar to canonical GSDMD-mediated pyroptosis.

GSDMD functions were identified in immune cells. Similar to GSDMD, GSDME presents the ability to form membrane pores. Unlike GSDMD, GSDME functions need to be deeply clarified in immune cells through more studies [32]. Recently, Zhang et al. [33] reported that GSDME facilitates tumor-associated macrophage to phagocytize tumor cell. It also increases the number and function of CD8^+^ and natural killer T lymphocytes in tumor microenvironment. Our study demonstrates that GSDME might restrain UVB-induce inflammation depending on its function in immune cells.

Previous studies reported that neutrophils are major proportion of immune cells infiltrating in skin exposed solar simulated radiation [34]. Infiltrating neutrophils are important source of photoaging-associated active enzymes, including MMP-9 and elastase [34]. Strickland et al. found that TNF-α and IL-8 are increased in epidermis of UVB-challenged human skin, and their upregulation is correlated with neutrophils accumulation [35]. Recently, neutrophils were found to migrate into not only skin tissue but also kidney tissue, leading to neutrophil-associated kidney inflammation [36]. Here, GSDME is involved in neutrophils function in the UVB-caused inflammatory microenvironment. Although we observed immune microenvironment for recruiting neutrophils in UVB-challenged *Gsdme*^−/−^ mice, the mechanism by which GSDME deficiency promotes infiltration and activation of neutrophils remains to be elucidated. Chen et al found that GSDME facilitates neutrophils pyroptosis and IL-1β release. In the process of *Yersinia pseudotuberculosis* infection, GSDME and likely itself in neutrophils mostly contribute to IL-1β release [37]. However, GSDME deficiency did not suppress neutrophils activity, but improved its role in UVB-induced skin inflammation. Therefore, the role of neutrophils GSDME in UVB-induced skin inflammation needs to be clarified.

We reported that pyroptosis executor GSDME plays a restricted role in UVB-induced skin inflammation, as its GSDME deficiency enhances the infiltration and activation of neutrophils. Although GSDME functions in immune cells remain unclear, infiltrating immune cells including neutrophils in UVB-challenged skin might participate inflammatory regulation through GSDME functions. Given that UVB-induced skin inflammation is the core mechanism in the pathogenesis of UV-associated skin disorders, GSDME function in immune cells recruited into UV-challenged skin should be deeply explored.

## Supplementary information

Supplemental material

Original Data File

Checklist

## Data Availability

All data generated during this study are included in this article. All data included in this manuscript may be requested from corresponding authors. For original data of western blots, please see the Supplemental Material titled original WB data.
