# Supplementary material for: GSDME deficiency leads to the aggravation of UVB-induced skin inflammation through enhancing recruitment and activation of neutrophils
Source: Cell Death Dis. 2022 Oct 1;13(10):841. doi: 10.1038/s41419-022-05276-9 (PMC9526747; doi:10.1038/s41419-022-05276-9)
Supplement: Supplementary file 1 — Supplemental material [file 41419_2022_5276_MOESM1_ESM.docx]

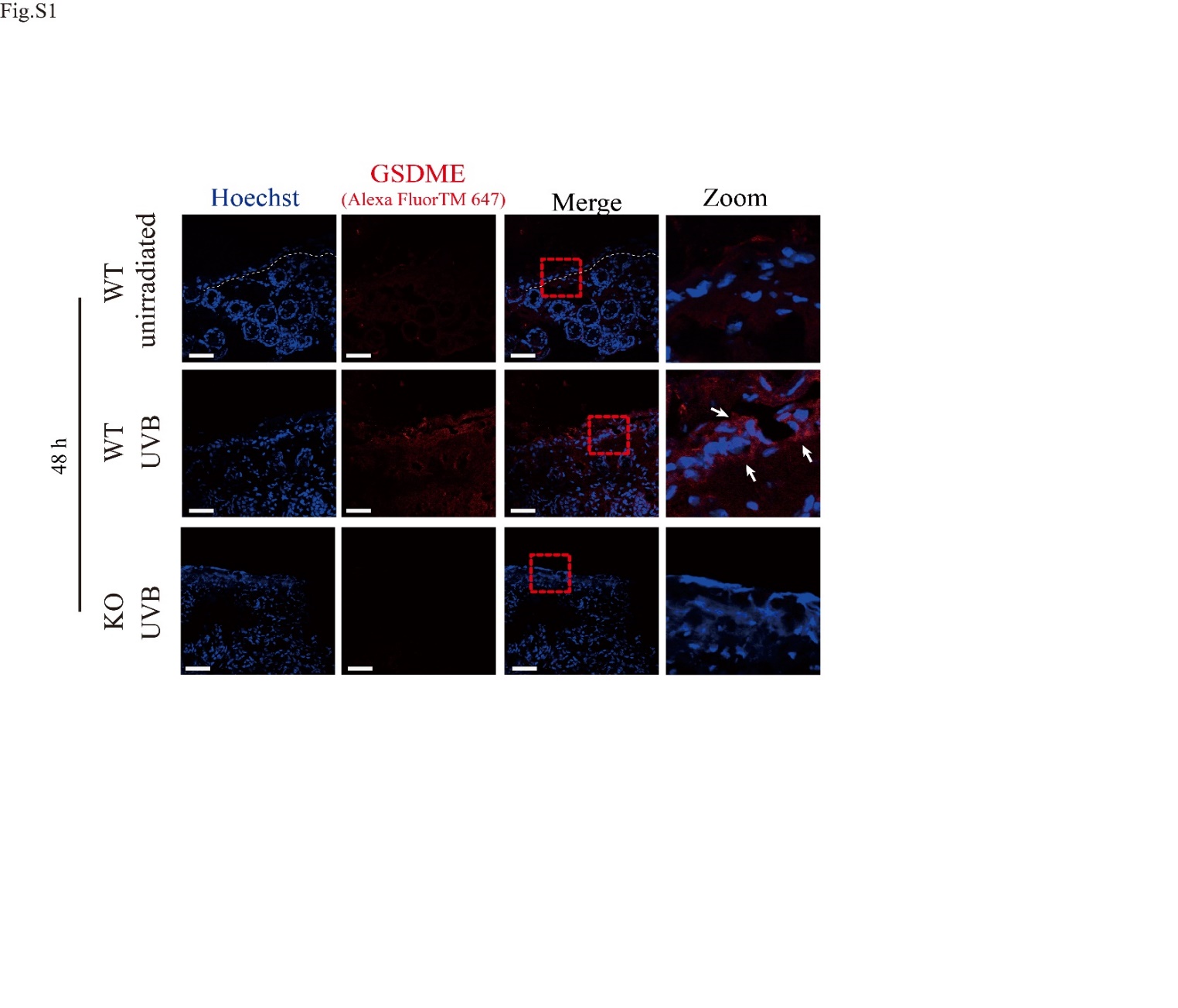


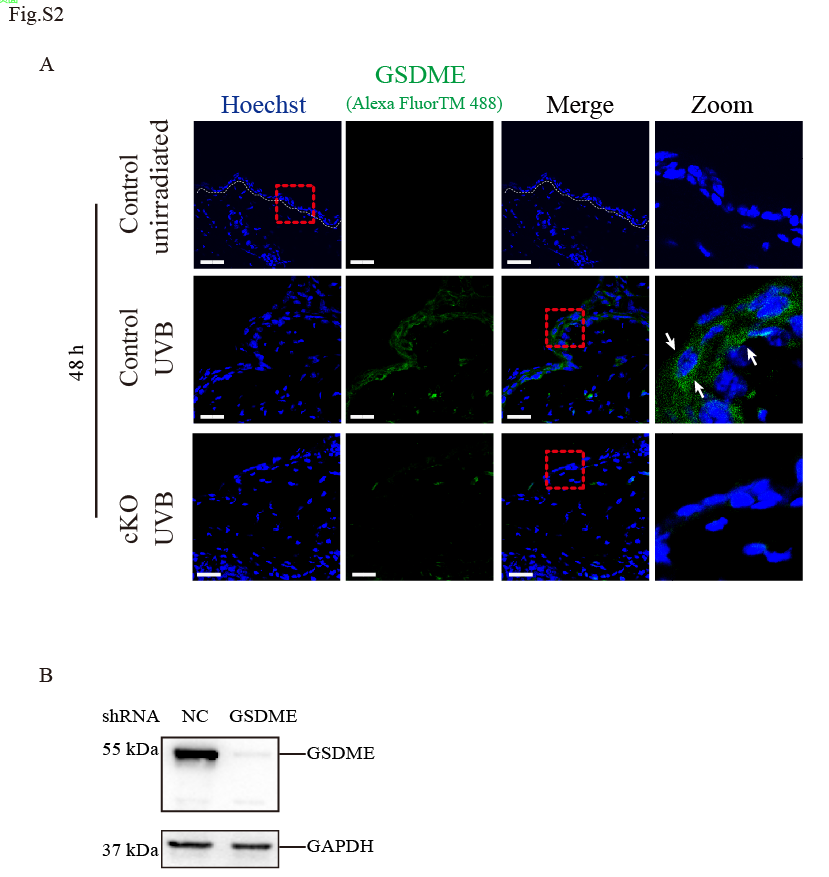


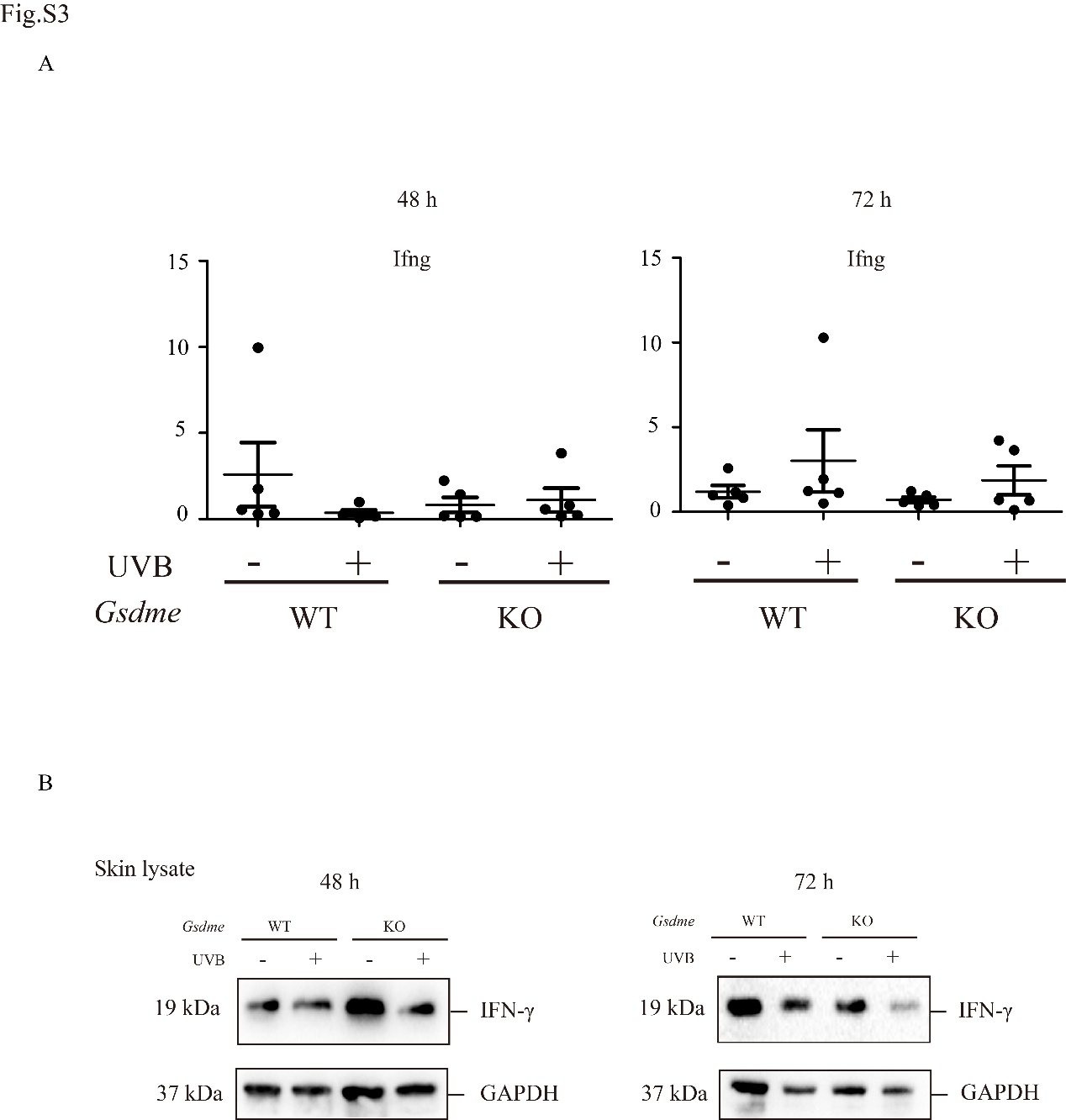

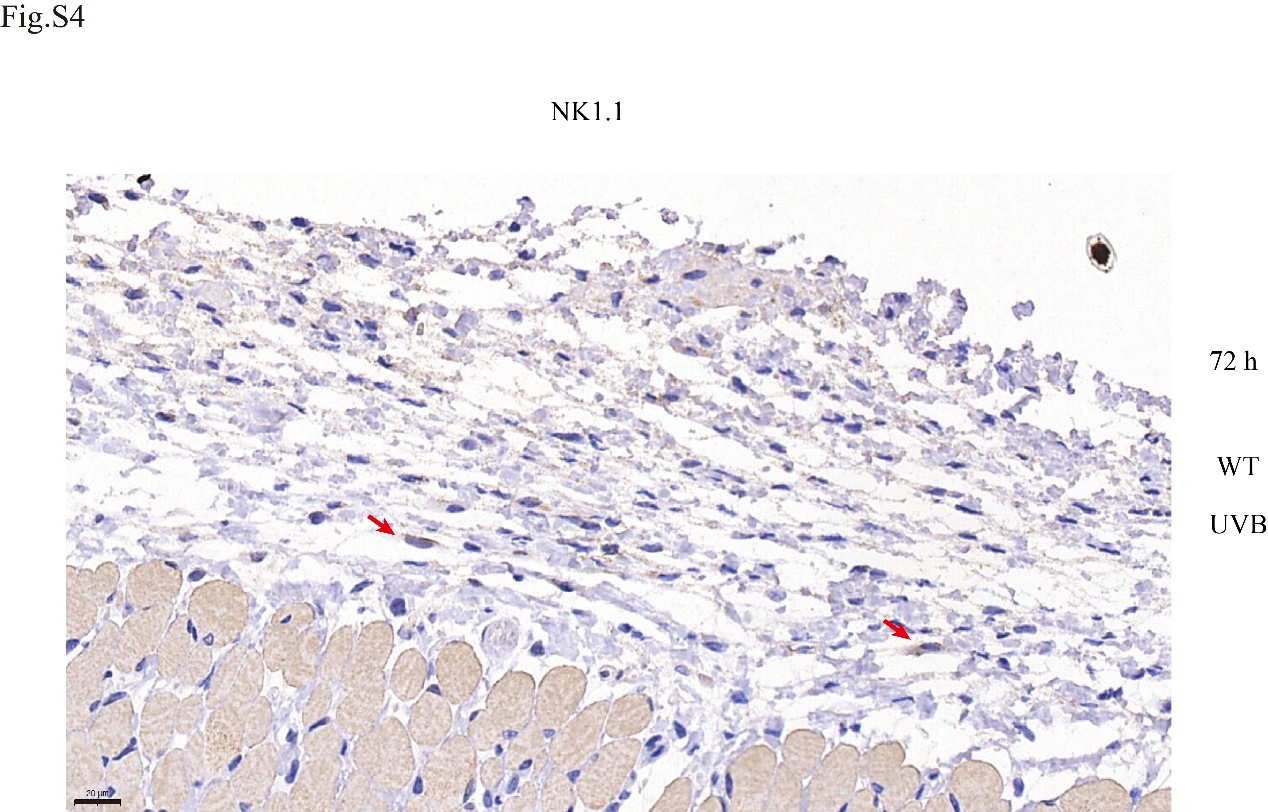


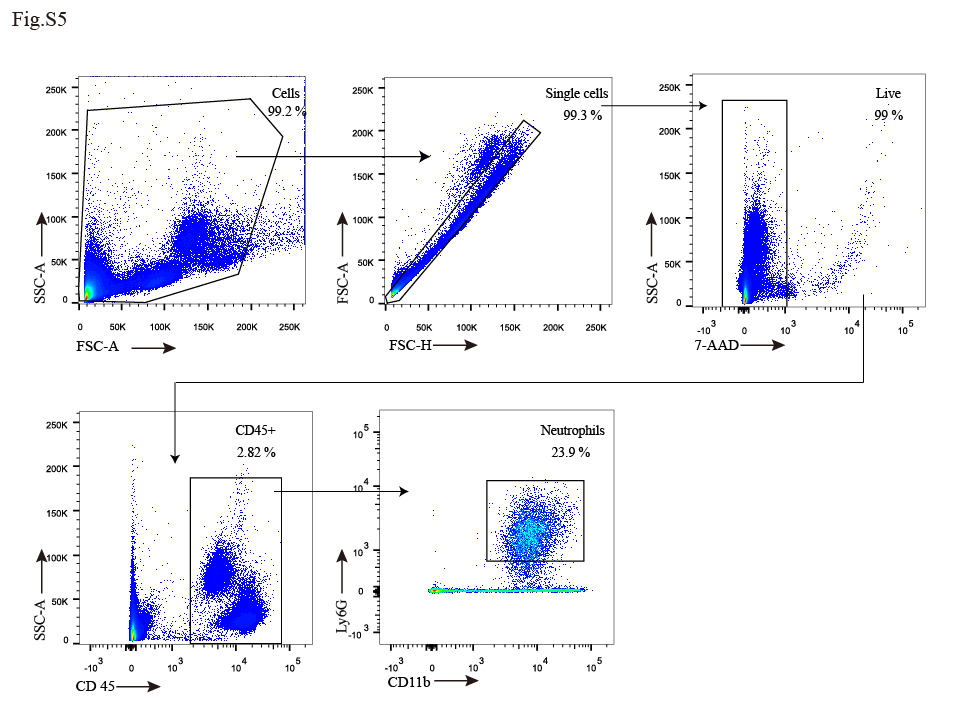


**Figure Legends**

**Fig.S1 Efficiency of GSDME knockout in mice**

The efficiency of GSDME knockout was detected by immunofluorescence assay (Alexa FluorTM 647, red). Scale bar represents 100 μm.

**Fig.S2 Efficiency of GSDME conditional knockout in mice and GSDME** **knockdown** **in HaCaT**

(A) The efficiency of keratinocyte-specific GSDME conditional knockout was detected by immunofluorescence assay (Alexa FluorTM 488, green). Scale bar represents 100 μm. (B) The GSDME knockdown efficiency in HaCaT transfected shRNA was validated by western blotting assay.

**Fig.S3 IFN-γ is not increased in skin tissue of UVB-challenged mice**

(A and B): WT mice or *Gsdme*^-/-^ mice were exposed or unexposed to 430 mJ/cm^2^ UVB radiation. (A): The mRNA levels were determined by Real-Time Quantitative Reverse Transcription PCR in the skin tissue at 48 and 72 h after UVB exposure. GAPDH served as the reference gene. (B): The protein levels of IFN-γ were detected by western blotting assay in the skin lysate at the same time points.

**Fig.S4 Detection of NK 1.1 in skin tissue of mice**

Red arrow indicates the NK 1.1 positive cells in skin tissue of WT mice at 72 h after UVB exposure radiation. Scale bar represents 20 μm.

**Fig.S5 Gating strategy for flow cytometry analysis of neutrophils (related to Fig. 6a)**

FSC-H/FSC-A was used to identify single cells. 7-AAD was used to identify live cells. The percentage of neutrophils (CD45^+^CD11b^+^Ly6G^+^) was calculated from CD45^+^ cells.
