# Supplementary figures and images for: GSDME deficiency leads to the aggravation of UVB-induced skin inflammation through enhancing recruitment and activation of neutrophils
Source: Cell Death Dis. 2022 Oct 1;13(10):841. doi: 10.1038/s41419-022-05276-9 (PMC9526747; doi:10.1038/s41419-022-05276-9)

Figure 1

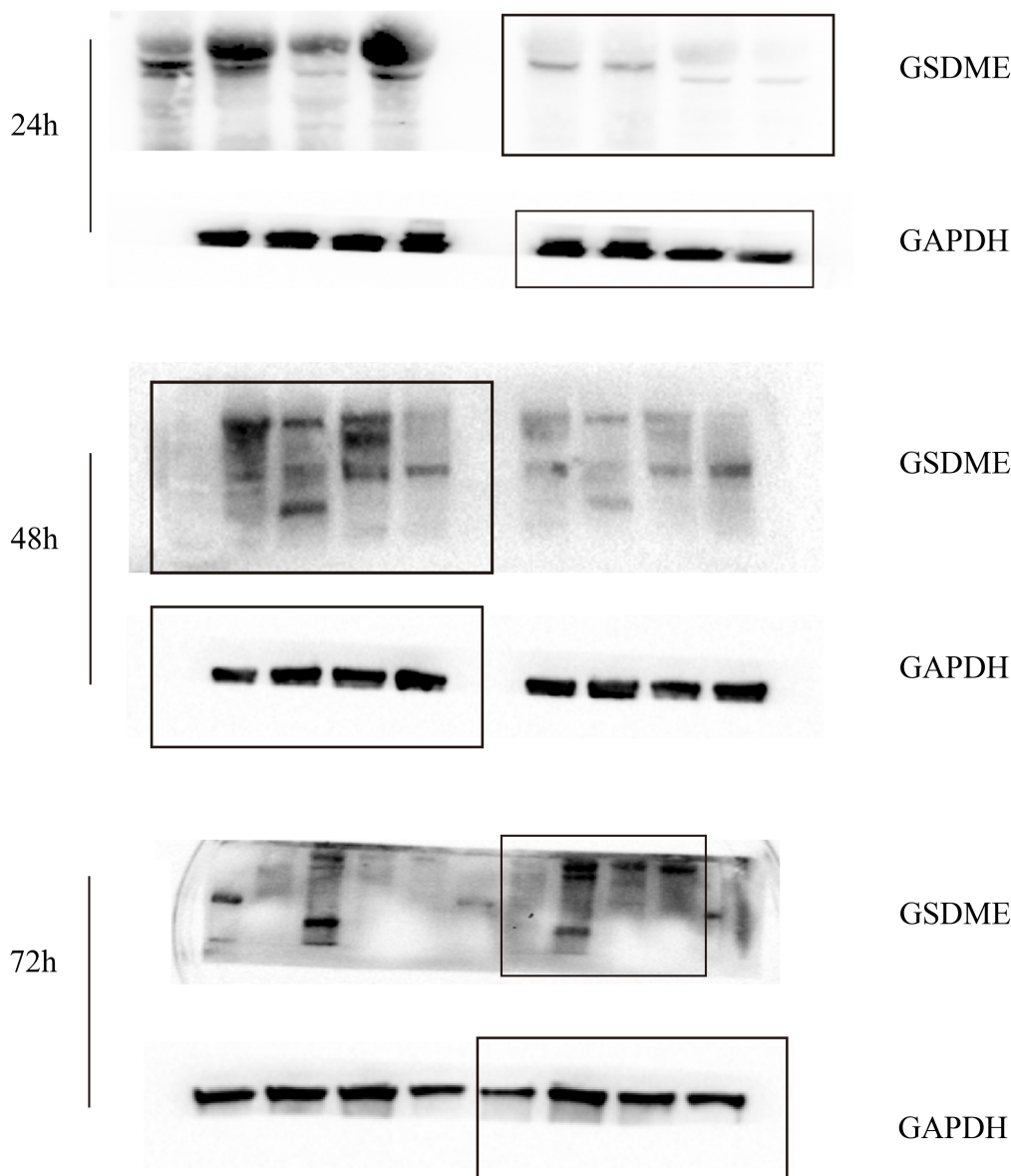

Figure 2

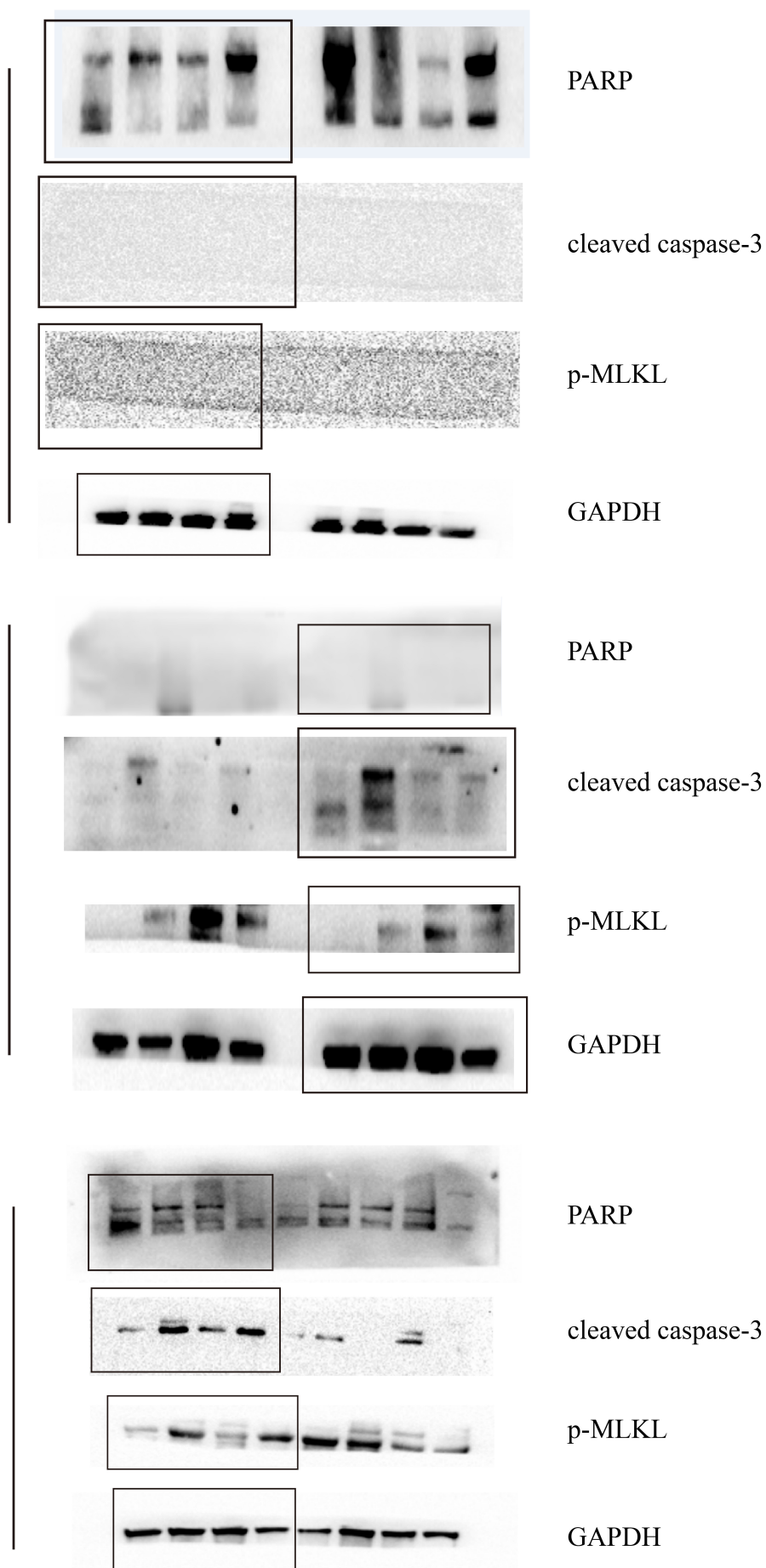

Figure 5

D

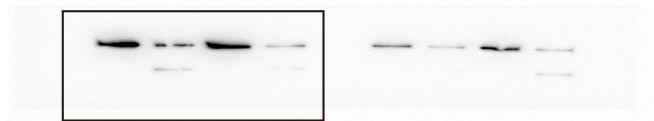

PARP

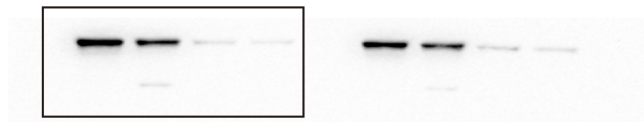

GSDME

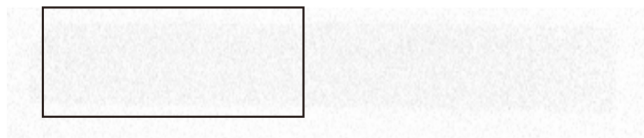

p-MLKL

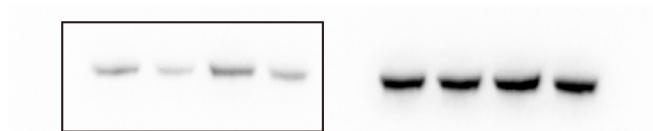

MLKL

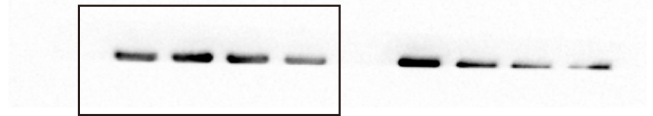

GAPDH

Figure S2 & S3

S2

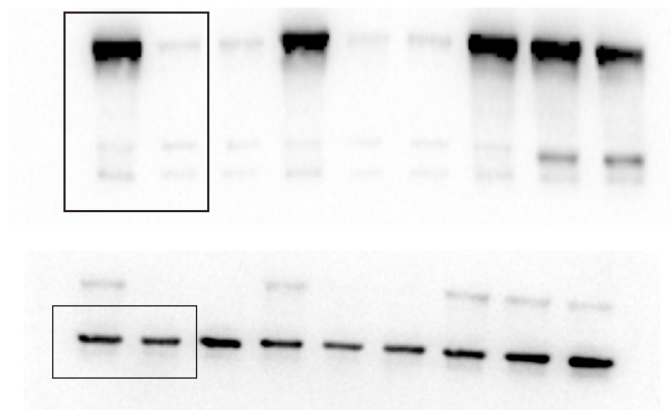

S3

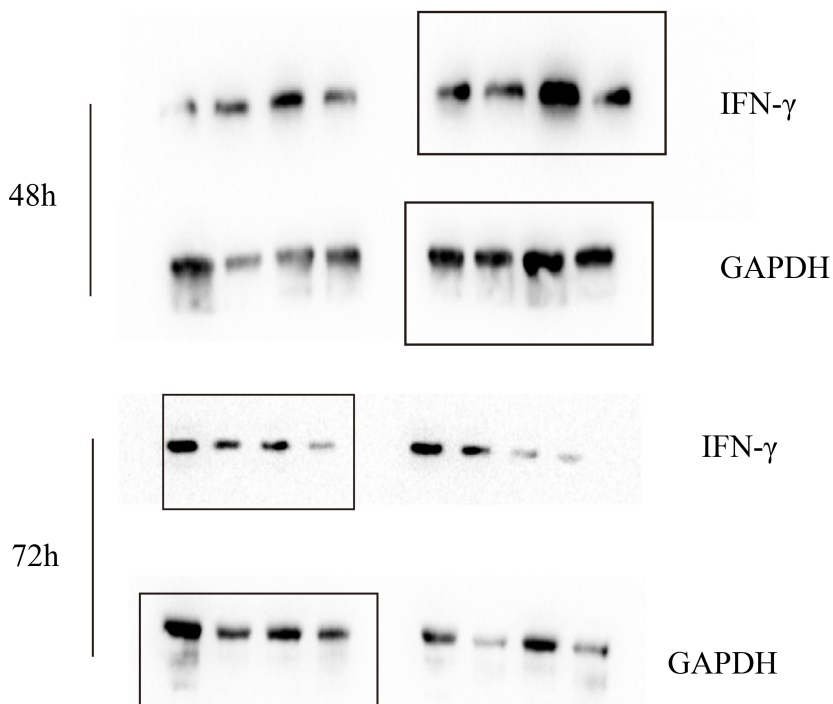

Supplement: Supplementary file 2 — Original Data File [file 41419_2022_5276_MOESM2_ESM.pdf]
